# Supplementary material for: Fast stimulated Raman and second harmonic generation imaging for intraoperative gastro-intestinal cancer detection
Source: Sci Rep. 2019 Jul 11;9:10052. doi: 10.1038/s41598-019-46489-x (PMC6624250; doi:10.1038/s41598-019-46489-x)
Supplement: Supplementary file 1 — Supp Info [file 41598_2019_46489_MOESM1_ESM.pdf]

Fast stimulated Raman and second harmonic generation imaging for intraoperative gastro-intestinal cancer detection

Barbara Sarri[1], Rafaël Canonge[1], Xavier Audier[1], Emma Simon[1], Julien Wojak[1], Fabrice Caillol[2], Cécile Cador[2], Didier Marguet[3], Flora Poizat[2], Marc Giovannini[2,*] and Hervé Rigneault[1,*]

[1]Aix Marseille Univ, CNRS, Centrale Marseille, Institut Fresnel, Marseille, France

[2]Institut Paoli-Calmettes, Endoscopy and Gastroenterology Departement, Marseille, France

[3]Aix-Marseille Université, INSERM, CNRS, Centre d'Immunologie de Marseille-Luminy, Marseille, France

*Corresponding authors: giovanninim@ipc.unicancer.fr, herve.rigneault@fresnel.fr

SUPPLEMENTARY INFORMATION

S1: λ-switch and FM-SRS modalities for SRH imaging

The developed λ-switch (Fig S1-1) and FM-SRS (FigS1-2) are three beams SRS modalities that allow to image two vibrational bonds sequentially within 1s and few microseconds with the λ-switch and FM-SRS modalities, respectively. The two targeted vibrational bonds are the carbon hydrogen CH_2 ($2845 cm^{-1}$) and CH_3 ($2930 cm^{-1}$) that are the signature of lipid rich and protein rich sample areas, respectively.

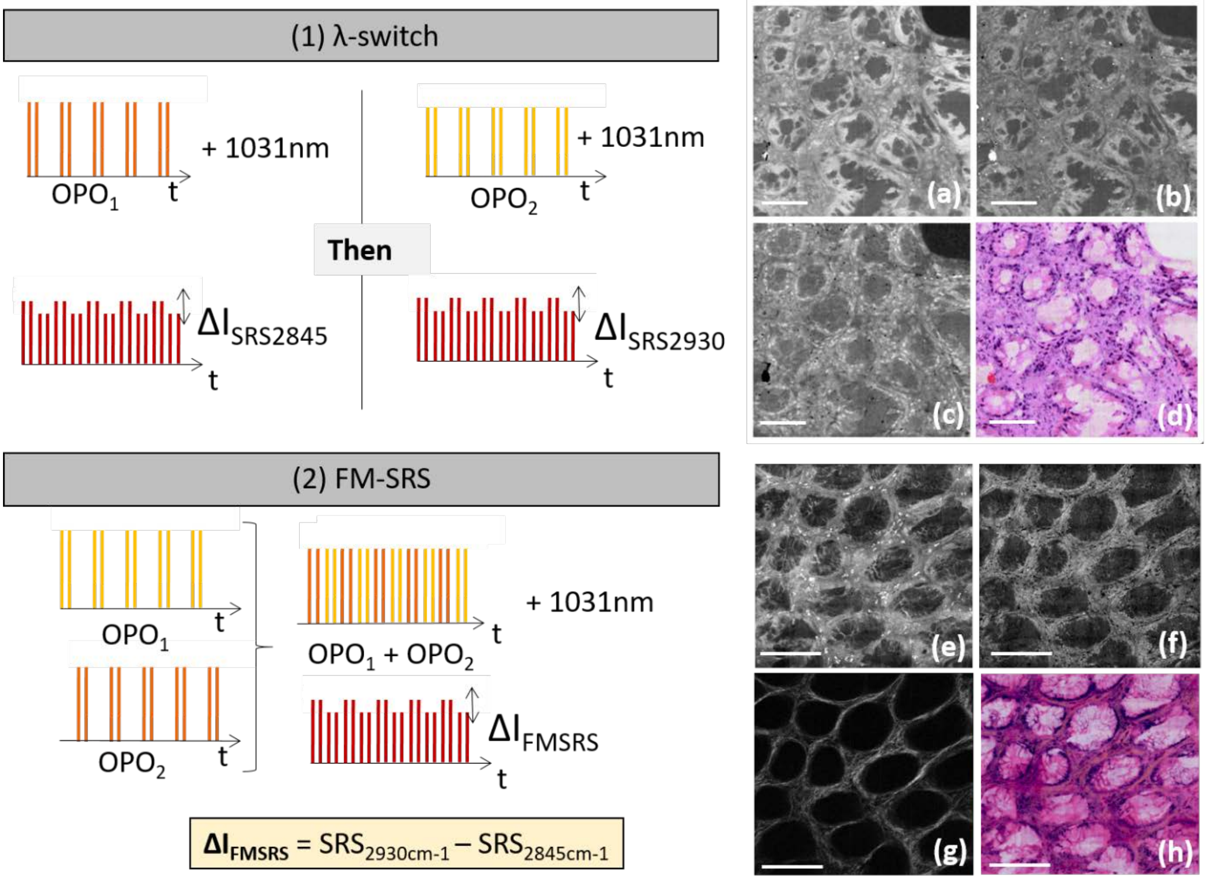

Fig S1 : λ-switch (1) and FM-SRS (2) modalities together with the generated raw images in the case of healthy colon tissues. λ-switch is a three beam SRS scheme that generates sequentially (within 1s) $SRS_{2845cm-1}$ (CH_2 bonds) (a) and $SRS_{2930cm-1}$ (CH_3 bonds) (b) while SHG is acquired simultaneously. The $SRS_{2845cm-1}$ and $SRS_{2930cm-1}$ images are subtracted to one another (c) and used to generate SRH images (d). FM-SRS is a three beam SRS scheme that generates simultaneously, CARS, TPEF (e), SRS_{nuclei}= $SRS_{2930cm-1}$ – $SRS_{2845cm-1}$ (f) and SHG (g). These images are used to generate SRH images (h). Scale bar 100 μm.

S2: Virtual HES coloring: Developed user interface

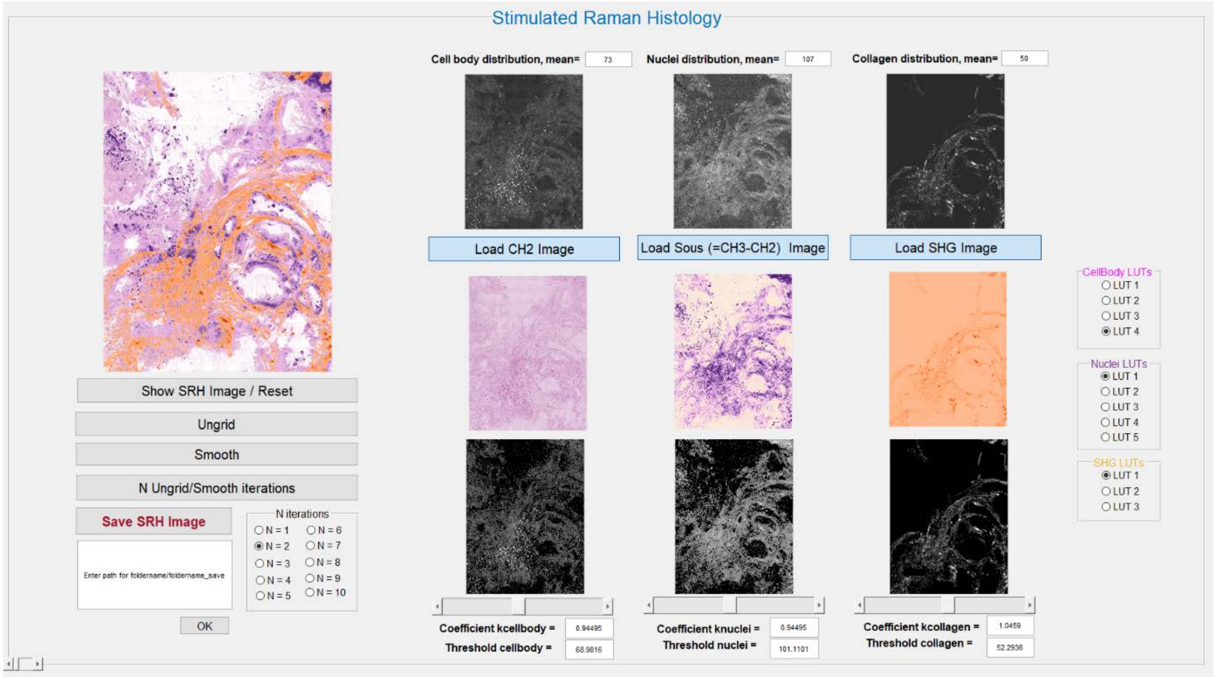

Fig. S2: Developed user interface for virtual HES colouring

S3: Virtual HES colouring example on a colon tissue section

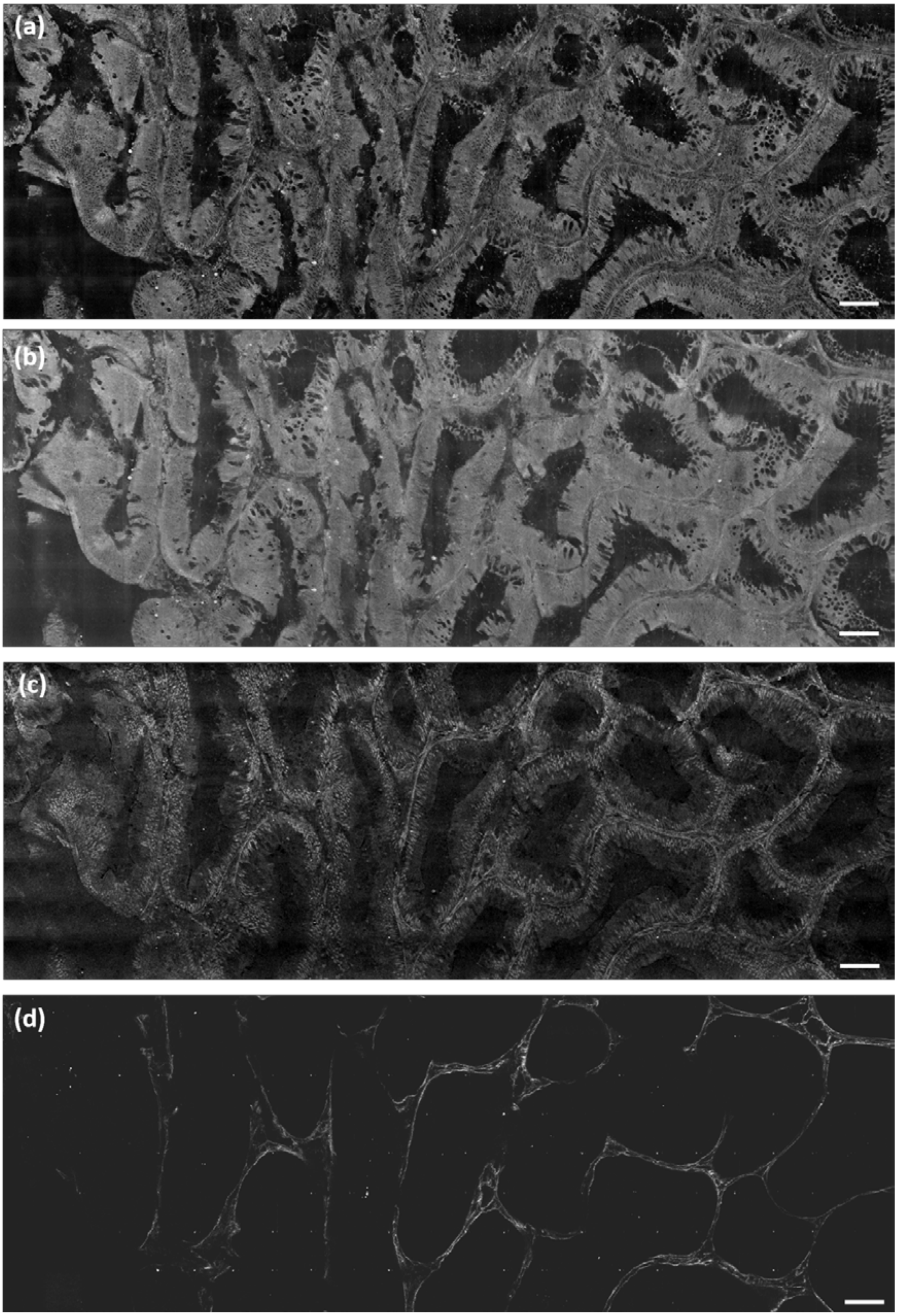

Fig S3 : Raw images in the case of a colon tissue section (low grade dysplasia). (a) $SRS_{2845cm-1}$ (CH_2 bonds) and (b) $SRS_{2930cm-1}$ (CH_3 bonds) stitched image are subtracted to build the (c) SRS_{nuclei}= $SRS_{2930cm-1}$ – $SRS_{2845cm-1}$ nuclei image while (d) SHG is acquired simultaneously. Scale bares 100 µm.

S4: SRH and HES images of healthy colon.

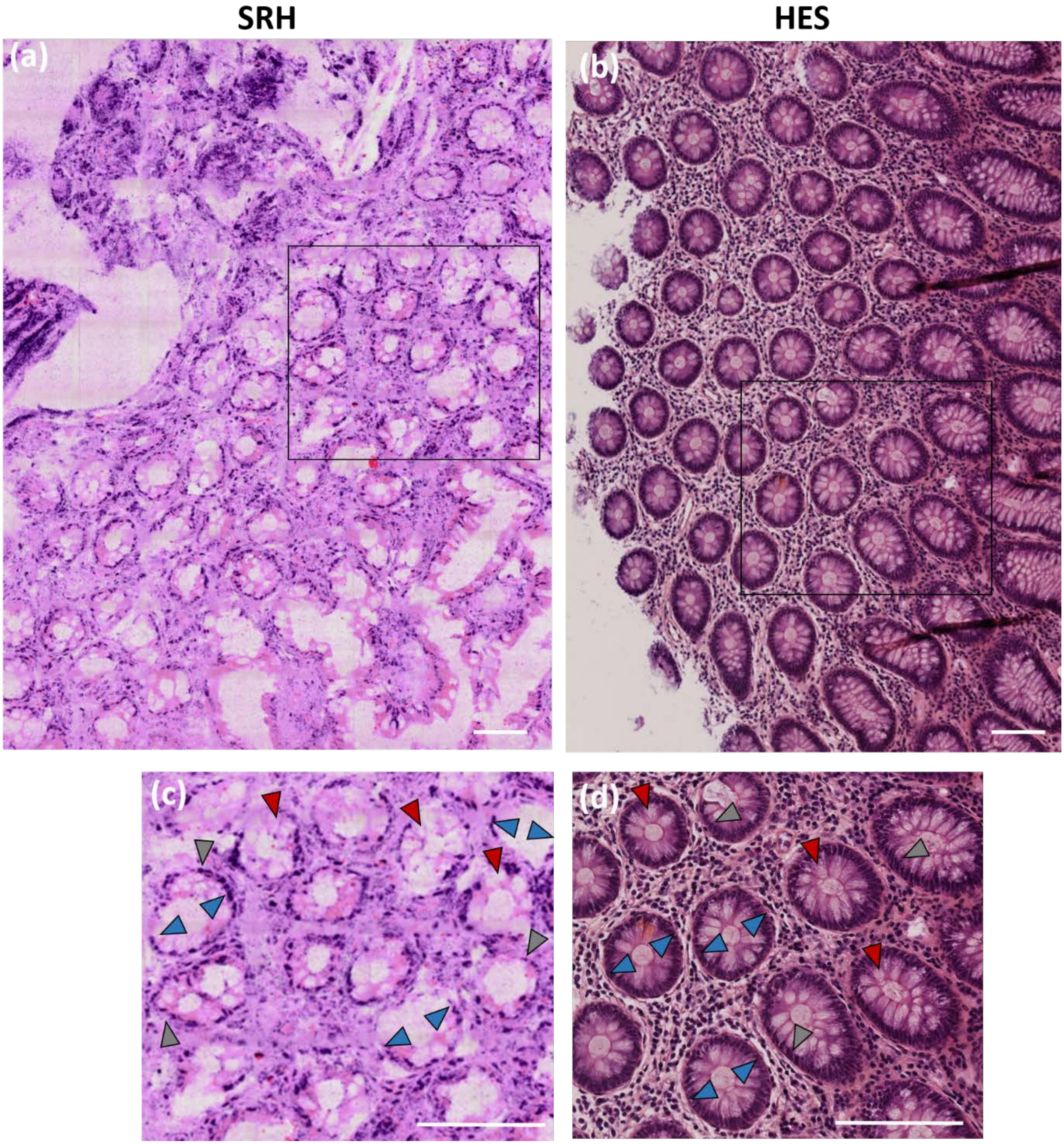

Fig S4: SRH (a) and HES (b) images of healthy colon over a 0.8 mm x 1 mm are compared. Zooms reveal that the SRH image (c) contains all the HES histology microscopic features (d) associated with a healthy colon sample: Homogeneous crypts diameters (blue arrows); nuclei regularly spaced around the glands (grey arrows), vacuoles uniformly present within the crypts (red arrows). Scale bares 100 µm.

S5 : SRH and HES images of colon low grade dysplasia

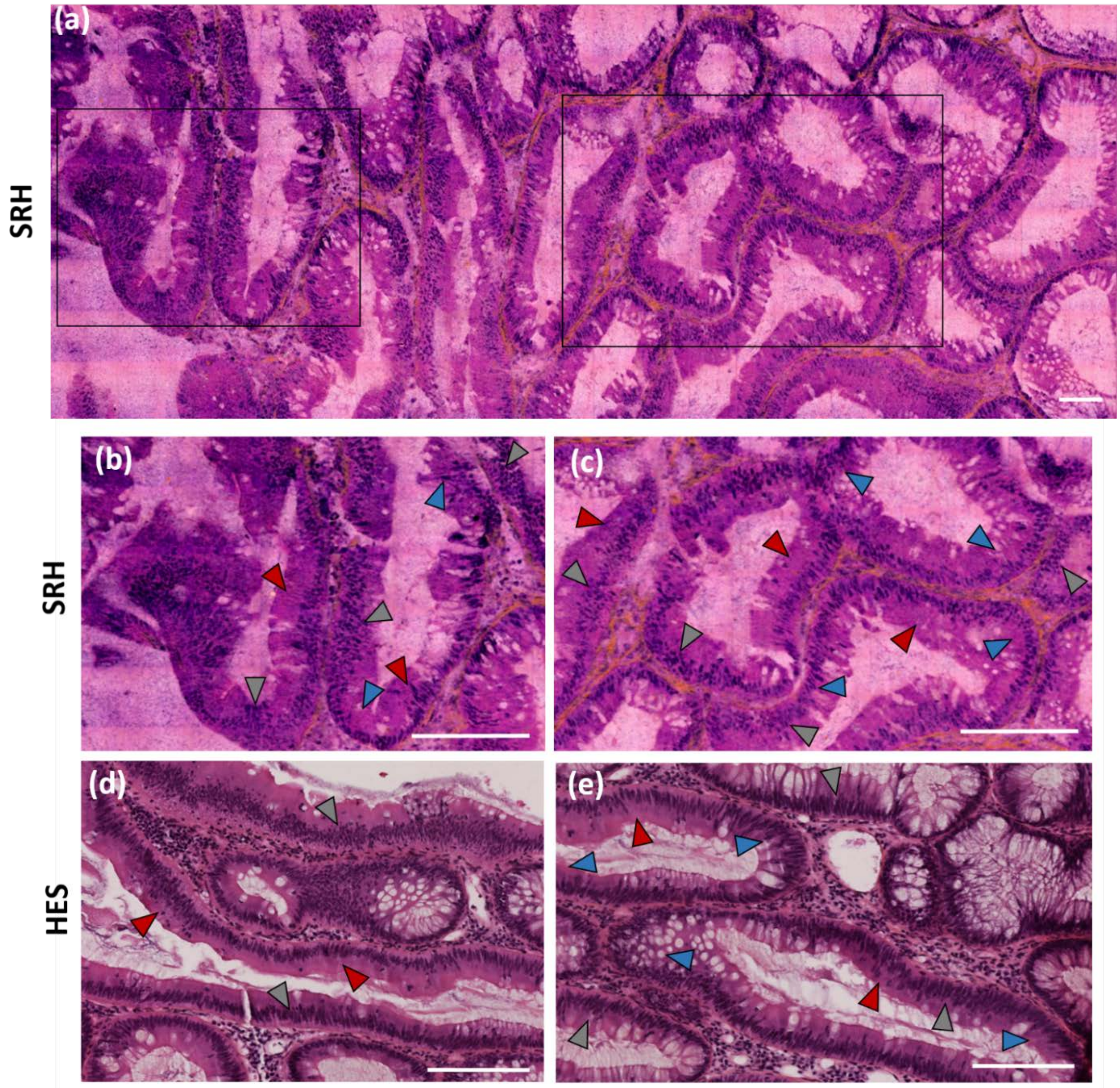

Fig. S5 : SRH (a) (b)(c) and HES (d) (e) images of colon low grade dysplasia are compared. The glands are larger with irregular and inhomogeneous shapes (bue arrows); lines of nuclei are present (grey arrows), and there is a reduction of the presence of vacuoles within the glands (red arrows). All these microscopic features seen on both SRH and HES images confirmed a low grade dysplasia diagnostic.

S6 : Composite SRS and toluidine blue images on healthy, cancerous and precancerous colon.

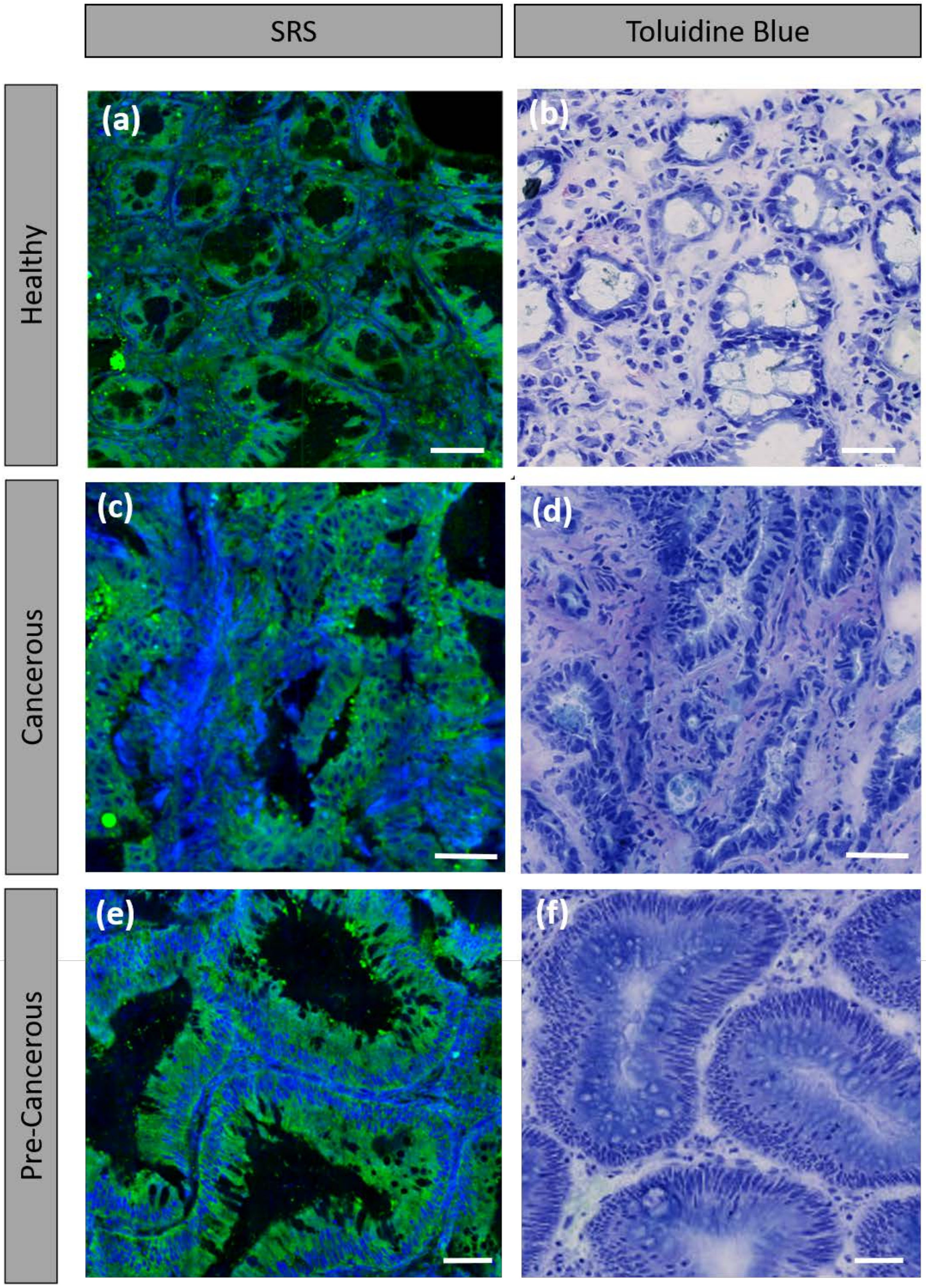

Fig. S6: SRS composite images revealing the nuclei (blue: CH_3 - 2930cm^{-1}) and the cell bodies (green: CH_2 - 2845cm^{-1}) of healthy (a), cancerous (c) and precancerous (e) colon tissues are compared to

toluidine blue images (b, d and f). Similar architectures are observed for nuclei and cell bodies in terms of shape, size and distribution. Scale bar 50 μm.

S7: SRH and HES images of healthy pancreas

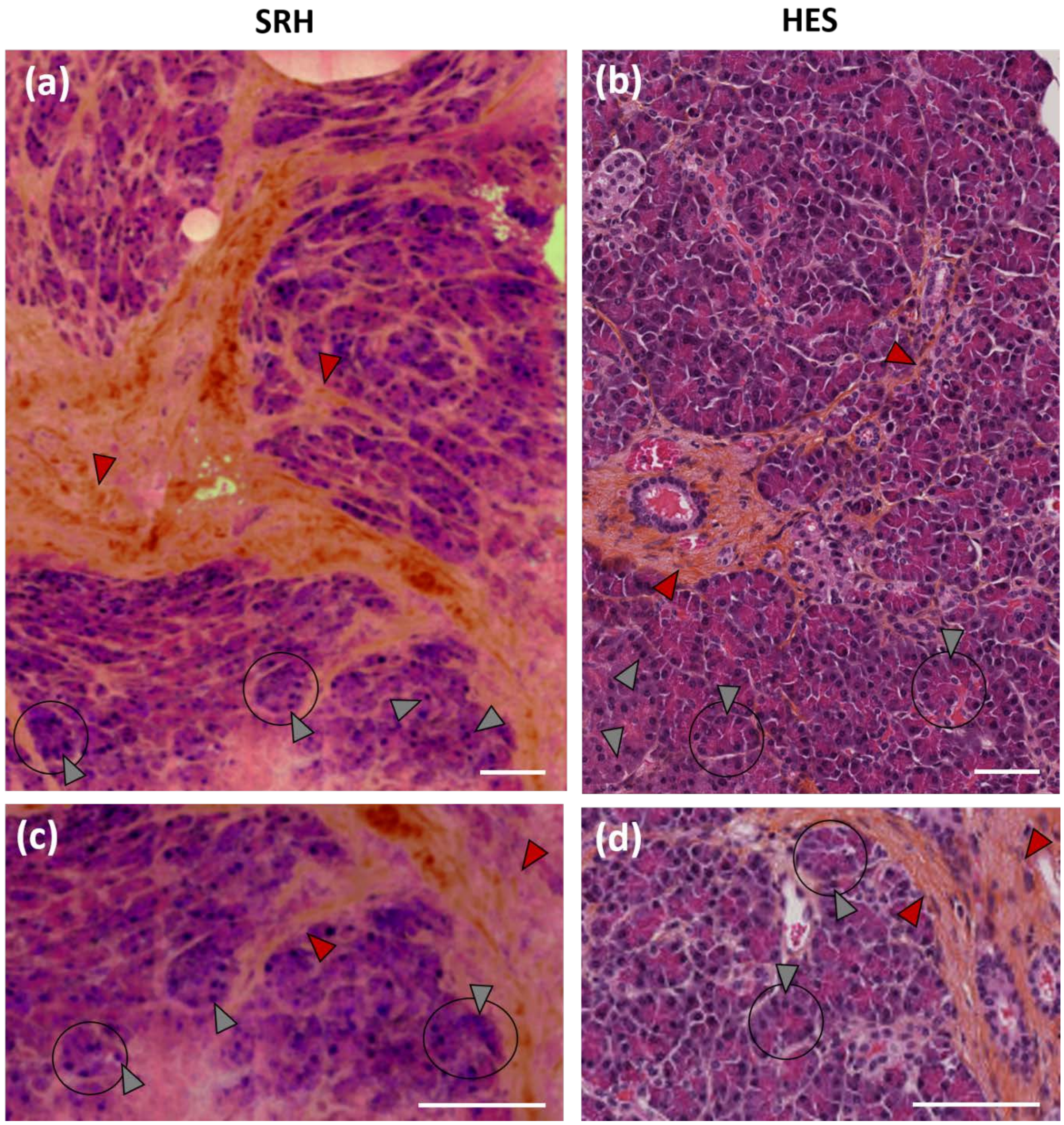

Figure S7 : SRH (a) (c) and HES (b) (d) images of healthy pancreas. Acini clusters (black circles) are surrounded by thin collagen fibres (red arrows). Nuclei within the acini are well defined in basal position (toward the edge of the cluster) (grey arrows) Scale bar is 100 μm.

S8 : FM-SRS image on a 1mm thick fresh colon tissue sample

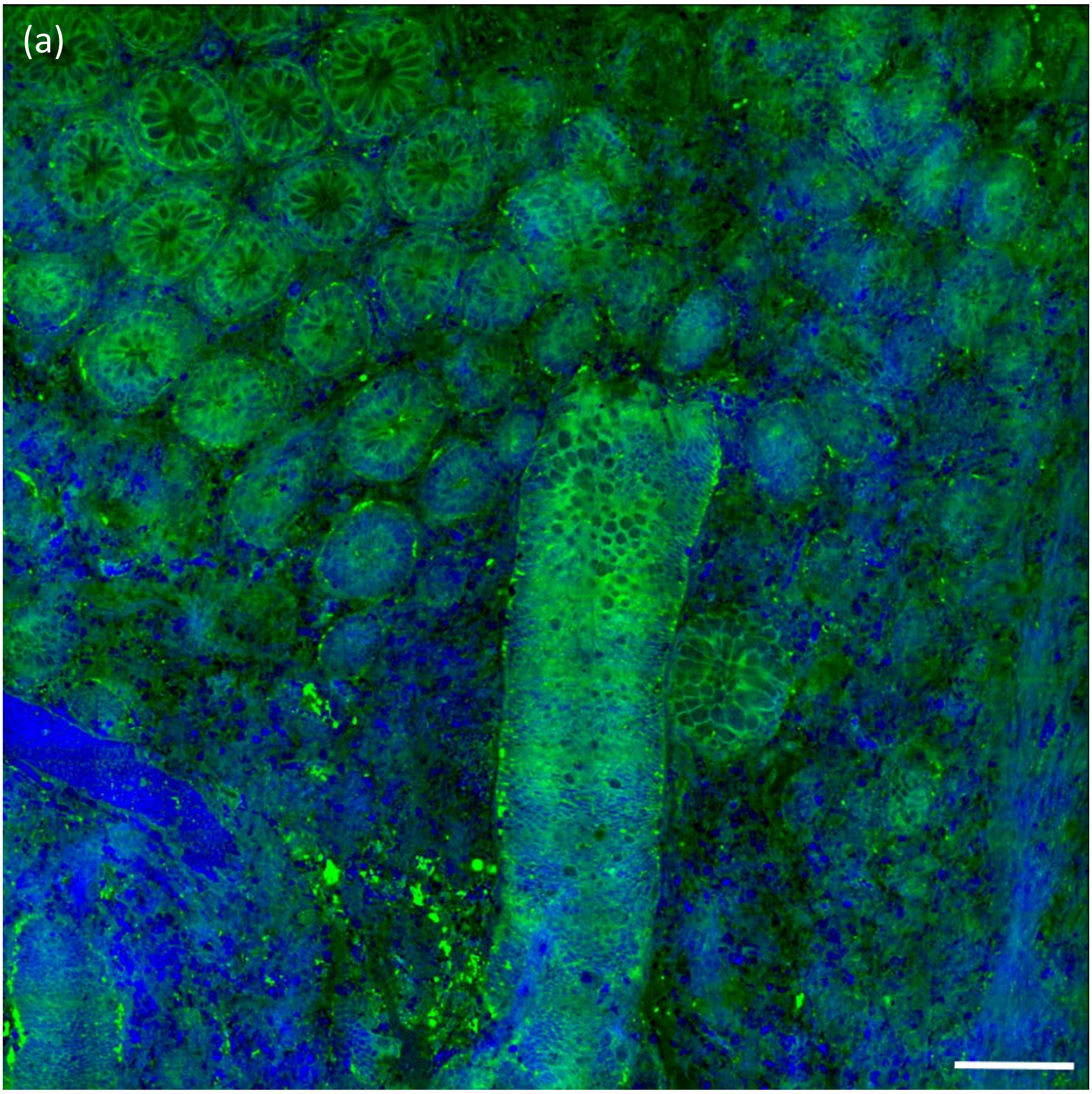

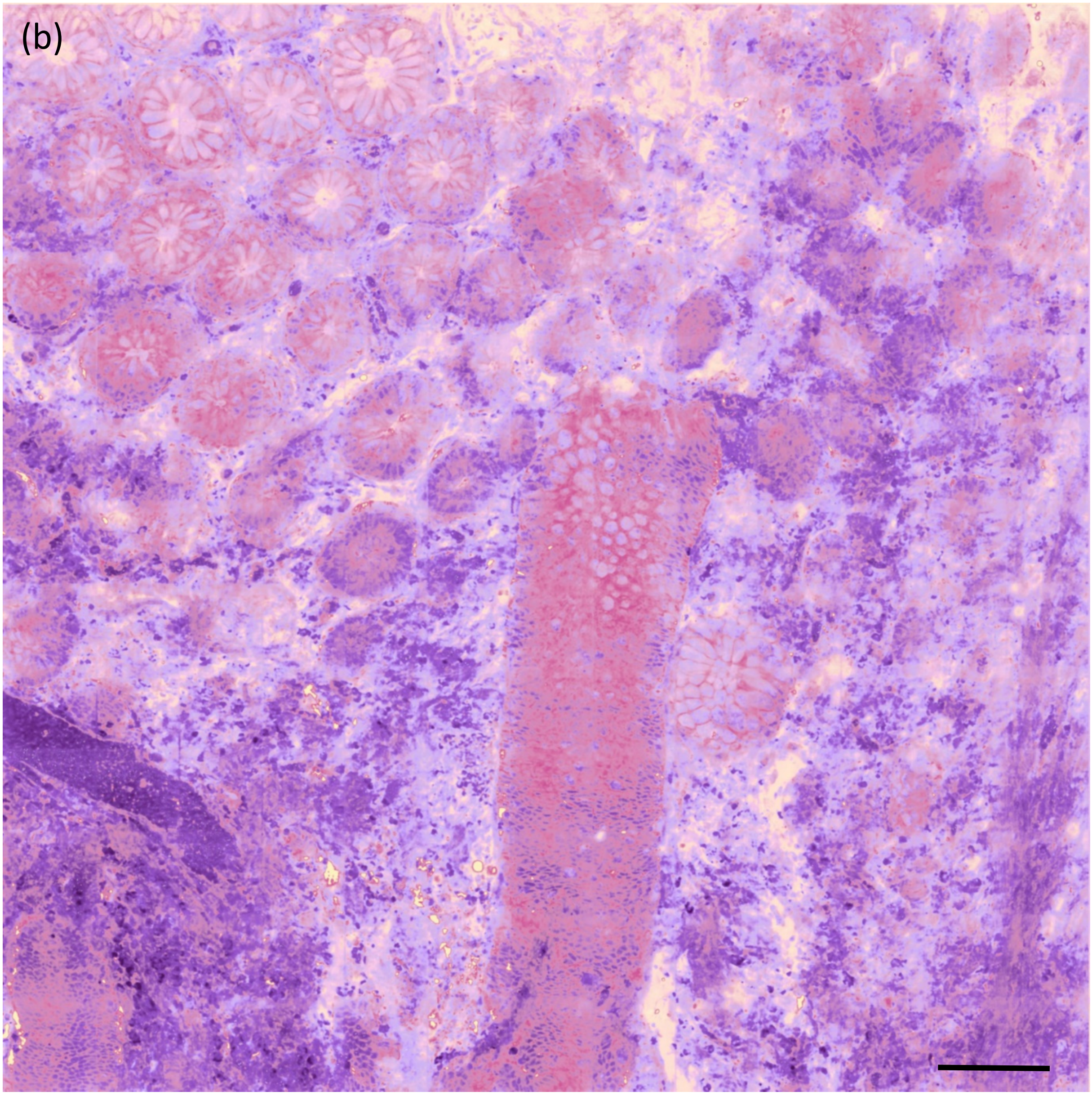

Figure S8 : (a) Composite FM-SRS (blue) and TPEF (green) and (b) SRH image from a 1 mm thick fresh healthy human colon sample. The image is performed at a depth of 50 μm below the tissue surface and the SRS detection performed in the forward direction (the laser are travelling through the tissue sample). Power laser at the sample is 40 mW from each beam. Total acquisition time is 25 min. Scale bar is 100 μm.
